# MiRNA and LncRNA as Potential Biomarkers in Triple-Negative Breast Cancer: A Review

**DOI:** 10.3389/fonc.2020.526850

**Published:** 2020-11-20

**Authors:** Simona Ruxandra Volovat, Constantin Volovat, Irina Hordila, Dorin-Alexandru Hordila, Ciprian Camil Mirestean, Oana Tatiana Miron, Cristian Lungulescu, Dragos Viorel Scripcariu, Cati Raluca Stolniceanu, Assia Adrianova Konsoulova-Kirova, Cristina Grigorescu, Cipriana Stefanescu, Cristian Constantin Volovat, Iolanda Augustin

**Affiliations:** ^1^ Department of Medical Oncology-Radiotherapy, Grigore T Popa University of Medicine and Pharmacy, Iași, Romania; ^2^ Center of Oncology Euroclinic, Iași, Romania; ^3^ Department of Pneumology, Hospital of Pneumophtysiology, Iasi, Romania; ^4^ Department of Medical Oncology, University of Medicine and Pharmacy, Craiova, Romania; ^5^ Department of Surgery, Grigore T Popa University of Medicine and Pharmacy, Iași, Romania; ^6^ Department of Biophysics and Medical Physics—Nuclear Medicine, University of Medicine and Pharmacy Gr. T. Popa Iasi, Iași, Romania; ^7^ Department of Medical Oncology, Complex Oncological Center, Burgas, Bulgaria; ^8^ Department of Radiology, Grigore T Popa University of Medicine and Pharmacy, Iași, Romania

**Keywords:** miRNA, LncRNA, biomarkers, ceRNA, circulating miRNA, triple negative breast cancer

## Abstract

Noncoding RNAs (ncRNAs) include a diverse range of RNA species, including microRNAs (miRNAs) and long noncoding RNAs (lncRNAs). MiRNAs, ncRNAs of approximately 19–25 nucleotides in length, are involved in gene expression regulation either via degradation or silencing of the messenger RNAs (mRNAs) and have roles in multiple biological processes, including cell proliferation, differentiation, migration, angiogenesis, and apoptosis. LncRNAs, which are longer than 200 nucleotides, comprise one of the largest and most heterogeneous RNA families. LncRNAs can activate or repress gene expression through various mechanisms, acting alone or in combination with miRNAs and other molecules as part of various pathways. Until recently, most research has focused on individual lncRNA and miRNA functions as regulators, and there is limited available data on ncRNA interactions relating to the tumor growth, metastasis, and therapy of cancer, acting either on mRNA alone or as competing endogenous RNA (ceRNA) networks. Triple-negative breast cancer (TNBC) represents approximately 10%–20% of all breast cancers (BCs) and is highly heterogenous and more aggressive than other types of BC, for which current targeted treatment options include hormonotherapy, PARP inhibitors, and immunotherapy; however, no targeted therapies for TNBC are available, partly because of a lack of predictive biomarkers. With advances in proteomics, new evidence has emerged demonstrating the implications of dysregulation of ncRNAs in TNBC etiology. Here, we review the roles of lncRNAs and miRNAs implicated in TNBC, including their interactions and regulatory networks. Our synthesis provides insight into the mechanisms involved in TNBC progression and has potential to aid the discovery of new diagnostic and therapeutic strategies.

## Introduction

Triple negative breast cancer (TNBC) represents a heterogeneous subgroup comprising 12%–17% of all of breast cancer (BC) (1). TNBC is characterized by the absence of estrogen receptor (ER), progesterone receptor (PR), and human epidermal growth factor receptor-2 (HER2), relative to normal tissue or other types of BC, as well as a high proliferative index (determined by mitotic or Ki-67 proliferative indices), high histological grade, and high rates of metastasis (2–4). TNBCs frequently undergo early metastasis to visceral organs and brain (5–7) and are more often diagnosed at an advanced stage. The treatment options for TNBC are limited, and the 5-year survival rate is 77%. Furthermore, this neoplasm is often diagnosed at a relatively young age and is a major cause of female mortality (8–10).

The molecular heterogeneity of TNBC has been recently mapped using messenger RNA (mRNA), microRNA (miRNA), DNA, and epigenetic analyses of data from The Cancer Genome Atlas (TCGA) (11). Six molecular subtypes of TNBC were identified, each displaying a unique biology (12, 13), including basal-like BL1 (with high Ki-67, NRAS, Myc, ATR, and BRCA expression), BL2 (high expression of MET, TP53, EGFR, and EPHA2), immunomodulatory (overexpression of genes involved in immune cell signaling), mesenchymal, mesenchymal stem-like (chemoresistant) (13, 14), and luminal androgen receptor (LAR). Among these molecular subtypes, the BL2 subtype has the worst prognosis, and LAR tumors are associated with the best overall survival (OS) rates.

To improve the survival of patients with TNBC, identification of predictive biomarkers is necessary to assess the risk of metastasis, the response to treatment, or even to develop new therapies. Of novel and potentially useful biomarkers, there is good evidence for close correlation of abnormal expression of noncoding RNAs (ncRNAs) and TNBC development and progression.

Only 2% of the human genome comprises protein-coding gene sequences although more than 75% of the human genome is transcribed. Hence, most transcripts are noncoding genome sequences, including ncRNAs (15, 16). These sequences have regulatory roles in eukaryote cells, in which ncRNA malfunction is important in neoplastic processes (16, 17). Current research focuses on mutations or epigenetic modifications at different levels of gene expression regulation; however, it was recently discovered that this process is also highly modulated post-transcriptionally through repression or enhancement of mRNA. ncRNA expression profiles of tumors can be used to discriminate between different types of cancer (17).

## MiRNA: Biogenesis and Functions

MiRNAs are short, single-stranded, ncRNAs (19–25 nucleotides) that account for 1%–5% of the human genome (28,000 mature miRNAs have been identified) and regulate at least 30% of protein-coding genes (18, 19). They are involved in the regulation of tumor growth, proliferation, differentiation, and apoptosis as oncogenes and tumor suppressors (17, 20).

There are several phases involved in the generation of mature miRNAs. First, a nucleotide sequence is transcribed by RNA polymerase II from intergenic or intron-coding regions, resulting in capped, polyadenylated transcripts named primary-microRNAs (pri-miRNAs). Next, the primary transcript is processed by Drosha and DGCR8, nucleic ribonucleases that are part of a multiprotein complex, the microprocessor complex, which can recognize the junctions between hairpin structures and single-stranded RNA. The resulting product is generated by cleaving the RNA and producing a hairpin-shaped intermediate of 70–100 nucleotides, known as precursor-miRNA (pre-miRNA) (21, 22). The pre-miRNA crosses the nuclear membrane into the cytoplasm through the exportin-5 channel, in which another ribonuclease, Dicer, further processes it into short, double-stranded RNA fragments whose strands separate, and the mature single-stranded molecule adheres to the RNA-induced silencing complex (RISC), which is an effector molecule comprising miRNA and specific proteins.

MiRNA molecules can repress mRNA in two ways. In the first model, the miRNA hybridizes to the 3′ untranslated region (UTR) of their target mRNA. The complex, comprising RISC and the hybrid RNA molecule then mediates the cleavage and degradation of the target mRNA. In the second model, the miRNA does not bind perfectly to complementary sites in the 3′ UTR of its target mRNA, resulting in formation of a complex with RISC that only blocks mRNA translation. Regardless of the mechanism involved, the result is a decreased quantity of the translated product of the gene encoded by the target mRNA (17, 23).

MiRNAs can also promote ribosome biogenesis, resulting in target gene repression (24). Further, miRNAs are implicated in various biological and pathological regulatory processes, including the most malignant phenotype processes (25).

The transcription and function of miRNAs can be altered by slight changes in the activities of proteins involved in this process (26, 27), epigenetic modifications, single nucleotide polymorphisms (SNPs) in miRNA sequences (28, 29), hereditary mutations in the DNA encoding the miRNA (30, 31), or somatic mutation in miRNA genes (32–34). Each specific discrepancy in miRNA processing represents an opportunity for intervening at the post-transcriptional level by regulating miRNA expression. Detection of interactions with other individual precursor molecules is crucial for the discovery of new regulatory mechanisms, and the description of miRNA-protein interactions is indispensable for understanding the complex pathways governing miRNA activity (35).

To date, studies have focused on specific miRNA profiles that intervene at different levels of the malignant process; however, the reasons why miRNAs are over- or under-expressed remain unclear although multiple hypotheses have been proposed (36).

### Types and Functions of miRNAs in TNBC

MiRNAs generate unique signatures that reflect the stage of development of cells and tissues of origin. A single miRNA can have multiple mRNA targets, and one mRNA can be the target of several miRNAs. Main miRNAs involved in TNBC are summarized in **Table 1**. From oncogenic miRNAs involved in TNBC, several are of major interest, including miR-21 and miR-135b. MiR-21 maps to chromosome 17 and is encoded by the *MIR21* gene. MiR-21 over-expression is anti-apoptotic and contributes to tumor cell growth. Further, miR-21 can target various mRNA transcripts, and high levels of its expressions are associated with poor patient prognosis in TNBC patients (77). MiR-21 also contributes to EMT and was linked to positive lymph nodes, advanced disease stage, and short OS, resulting in the suggestion that it represents a molecular prognostic and disease progression marker (39). Downregulation of miR-21 in BC stem cells results in reversal of EMT, followed by decreased levels of HIF1α and inhibition of migration and invasion (40). MiR-135b is highly expressed in BC, in which it stimulates cell growth and can promote proliferation, invasion, and migration via large tumor suppressor 2 (LATS2), which it downregulates in TNBC cell lines. The miR-135b-LATS2 axis also represents a potential therapeutic target in TNBC (42). Tumor suppressor miRNAs in TNBC are mainly represented by MiR-101, MiR-125, MiR-145, MiR-200, and MiR-655. MiR-101 is downregulated in TNBC. When its expression is in the normal range, it inhibits cell progression in TNBC and suppresses *MCL-1* expression, thereby increasing tumor sensitivity to paclitaxel (53). MiR-125 downregulates *MAP2K7* and inhibits EMT when expressed normally. Suppression of miR-125 is linked to poor prognosis and resistance to chemotherapy in patients with TNBC. Further, miR-125 can be considered a prognostic factor in TNBC (54). MiR-145 targets the oncoprotein ROCK1, thereby suppressing BC tumor growth and migration. This miRNA also inhibits apoptosis in response to cIAP1 over-expression in MDA-MB-231 TNBC cells. When miR-145 is downregulated, it has the opposite effect, being correlated with progression and metastasis. Further, miR-145 is a promising diagnostic tool, as it can be easily detected in the serum, as well as a therapeutic target (56, 57). MiR-199a-5p is downregulated in TNBC, in which it has a tumor-suppressor role. High levels of miR-199a-5p repress invasiveness and migration in vitro, and its downregulation has a major impact on tumor growth and invasiveness. MiR-199a-5p is a reliable diagnostic marker and can be processed from blood plasma (60). The miR-200 family comprises MiR-200a, MiR-200b, Mir-200c, MiR-141, and MiR-429 with tumor-suppressor effects on apoptosis and metastasis of cell lines in vitro. In vivo, this family exhibits significantly reduced expression in metastatic TNBC. Levels of miR-200 miRNAs are decreased in mesenchymal subtype TNBC cell lines. Moreover, low expression of this family induces EMT through upregulation of ZEB1 (61, 62) and contributes to TNBC pathogenesis via multiple pathways (63). MiR-200a suppresses the EPH receptor A2 (*EPHA2*). MiR-200a is downregulated in basal-like BC, leading to elevated *EPHA2* expression and consequent poor patient OS (64). MiR-200b-3p and MiR-200b-5p are involved in a dual mechanism of EMT regulation, via targeting of ZEB1/2 and suppression of PKCα (65). MiR-200c downregulation may be involved in invasion and metastasis in TNBC cases with BRCA mutation (66). MiR-655 is also downregulated in TNBC, being correlated with lymph node metastasis. Furthermore, there is a correlation between the level of miR-655 and BC molecular classification. MiR-655 over-expression can downregulate EMT by suppression of PRRX1, inhibiting cell migration and invasion (74).

**Table 1:** Summary of the cellular functions of miRNAs in tumorigenesis of TNBC.

MiRNAs	Expression in TNBC	Mechanism	Biological function in TNBC	References
MiR-9	up	Inhibition of E-cadherin production, activation of β-catenin signaling	Rapid relapse, distant metastasis	(37, 38)
MiR-21	up	targeting mRNA transcripts, enhancing EMT	enhancing migration and invasion, short overall survival	(39, 40)
MiR-122	up	downregulating *PKM* gene, reprograms tumor cell glucose consumption	enhancing metastasis	(41)
MiR-135b	up	direct regulation of the expression of LATS2 and the Hippo pathway	promote proliferation, invasion and migration	(42)
MiR-146a miR-146b‐5p	up	repress BRCA1	promote proliferation, low overall survival rates	(43)
MiR-155	up	Inhibits VHL expression and induces angiogenesis	metastasis, poor prognosis	(44)
MiR-181	up	Stimulates activin and TGFβ growth factors	Decreased overall survival	(45)
MiR-182	up	downregulates profilin 1 (*PFN1*) gene expression	proliferation and invasion	(46)
MiR-221 MiR-222	up	regulation of uPAR directly isoforms 1,2,3,4	Metastasis, poor prognosis	(47, 48)
MiR-1	down	Target SLUG, upregulation inhibits MALAT	proliferation, tumor progression, and metastasis	(49)
MiR-26	down	modulates apoptosis and autophagy	positive lymph nodes, poor survival rates	(50)
MiR-31	Down	modulates *WAVE3* by short hairpin RNA	distal metastasis	(51)
MiR-34 family	Down	regulates SIRT1, p53 activity and NOTCH2	regulates cell cycle progression, cellular senescence, and apoptosis	(52)
MiR-101	Down	targets *MCL-1* expression	Increase tumor sensitivity to paclitaxel	(53)
MiR-125	Down	regulates MAP2K7 and EMT	poor prognosis, resistance to chemotherapy	(54)
MiR-136	Down	modulates EMT	Inhibits invasion, migration, correlated with tumor grade	(55)
MiR-145	down	targets ROCK1, inhibit apoptosis	progression and metastasis	(56, 57)
MiR-195	down	targets WNT3A expression	chemoresistance to doxorubicin, correlates with advance stage	(58, 59)
MiR-199a-5p	down	modulates stem-cell like and EMT	tumor growth and invasiveness	(60)
MiR-200	down	regulation of ZEB1/2 and PRKCA	invasion and metastasis in TNBC cases with BRCA mutation	(61–66)
MiR-203	down	targets BIRC5 and LASP1	correlated with lymph node metastasis and poor survival	(67–69)
MiR-205	down	major regulator of stemness and ZEB1 expression	associated with chemoresistance	(36, 70, 71)
MiR-206	down	targets Connexin 43, targets Coronin 1C	Proliferation, invasion, metastasis	(72)
MiR-638	down	BRCA1 tumors	correlated with overall survival	(73)
MiR-655	down	Modulates EMT, PRRX1	correlated with lymph node metastasis	(74)
MiR-1296	down	Modulates Cyclin D1	Sensitivity to cisplatin	(75)
Let-7	down	Regulation of HMGA2 Modulation of transcriptional repressor Blimp-1	Drug sensitivity	(76)

VHL, von Hippel-Lindau tumor suppressor; uPAR, urokinase-type plasminogen activator receptor; PRKCA, protein kinase C Alpha; HMGA, high mobility group 2A.

## Long Noncoding RNAs (lncRNAs): Biogenesis and Function

LncRNAs are a category of ncRNA transcripts that regulate gene expression at the transcriptional, translational, and post-translational levels; however, lncRNAs do not encode peptides or proteins although they are vital for the proper functioning of cellular processes (78). LncRNAs exert their functions through various mechanisms, including mediation of interchromosomal interactions, acting as sponges for endogenous RNAs, regulating mRNA decay, and modulating epigenetic components, which are redirected to their targets, among others. Hence, any change in lnRNA expression levels can lead to various illnesses, including malignant disease (15).

The definition and classification of lncRNAs remains somewhat unclear as the exact mechanisms and pathways of action of these molecules have not been fully elucidated although some classes of lncRNA have been intensively studied and no longer represent a mystery.

LncRNAs are >200 nucleotides long with the majority ranging from 1000 to 10,000 nucleotides. In addition, this type of ncRNA generates secondary and three-dimensional structures, allowing it to play dual RNA and protein-like roles (79, 80). LncRNA biogenesis presents some similarities with that of mRNA; it is transcribed by RNA polymerase II, after which the majority of transcripts are spliced. Unlike mRNAs, lncRNAs are predominantly localized in the nucleus and have lower expression levels. Moreover, lncRNA expression patterns are cell type–specific (79, 81).

Most lncRNAs localize to the nucleus and chromatin, where they control DNA sequences and are involved in transcriptional regulation with different functions in the cytoplasm, and a fraction of molecules occur as circulating lncRNAs, which are transmitted via exosomes (78).

LncRNAs can interact with various molecules, including transcription factors, mature mRNAs, chromatin-modifying complexes, RNA binding proteins, DNA, nascent RNA transcripts, microRNA, and chromatin. LncRNA transcripts can bind to active proteins and establish an exact position (*cis* or *trans*) (80). Consequently, lncRNAs have important functions in regulating gene expression at the epigenetic, transcription, and post-transcription levels (82).

### Types and Functions of lncRNAs in TNBC

It is established that dysregulation of lncRNAs contributes to the pathogenesis of cancers (78). Four different archetypes of lncRNA molecular functions have been described, proposing that these molecules can function as decoys, signals, scaffolds, and guides. LncRNAs can act as molecular signals because transcription of an lncRNA occurs at an exact moment and location to integrate developmental signals, interpret cellular context, or respond to diverse stimuli. In this archetype, lncRNAs can act as markers of functionally significant biological events. In the second archetype, lncRNAs function as a molecular decoy; in this context, following their transcription, lncRNAs bind to and titrate their targeted protein. Frequently lncRNAs act as negative regulators of effector molecules. The third lncRNA archetype is the guiding type. LncRNAs can act as gene expression guides in two ways: For genes situated in their immediate vicinity, they act in a *cis* form, and they are in a *trans* form for distant genes. LncRNAs bring together components of different complexes and transcription factors that can either repress or activate targeted genes. In the fourth archetype, lncRNAs act as scaffolds. Different lncRNA domains bind distinct effector molecules simultaneously, resulting in either activation or repression of transcription. A better understanding of how the scaffolding complexes are assembled and regulated provides potential new strategies to select and utilize precise signaling constituents to modify cellular activities. These four archetypes of lncRNA molecular functions involve numerous lncRNAs in various pathways specific to TNBC. In a fifth archetype, represented by competing endogenous RNAs (ceRNAs), lncRNAs act as molecular sponges for microRNAs, controlling their bioavailability with functional biological consequences at the cellular or physiological level (82).

Several lncRNAs have important roles in cancer development, some of which have also been identified in TNBC (**Table 2**). From oncogenic lncRNAs, the main important ones are HOTAIR, MALAT1, HULC, AWPPH and ARNILA. HOTAIR (Hox transcript antisense intergenic RNA) is one of the best-studied lncRNA regulators. It is a spliced, polyadenylated transcript comprising 6 exons; is approximately 2200 nucleotides in length; and maps to chromosome 12q13.13 (83). This sequence has roles in the progression of multiple neoplasms, including pancreatic, colorectal, hepatocellular, gastric, lung, ovarian, and BCs. HOTAIR is oncogenic when upregulated; hence, its expression levels are correlated with patient prognosis, and HOTAIR can be used as a predictive biomarker.

**Table 2:** Summary of the cellular functions of miRNAs in tumorigenesis of TNBC.

LncRNAs	Role in TNBC	Mechanism	Biological function in TNBC	References
HOTAIR	oncogenic	Remodeling chromatine, modulate PRC2	poor survival rates, marker of metastasis, chemoresistance	(83–93)
MALAT1	oncogenic	PI3k/AKT/mTOR and Wnt/β-catenin pathways sponge for miR-129-5p	proliferation, progression, metastasis	(94–98)
HULC	oncogenic	Regulation ofMMP-2 and MMP-9	Correlation with stage and overall survival	(99, 100)
AWPPH	oncogenic	upregulation of FZD7 association with miR-21	Correlate with tumor size, Involved in chemoresistance	(101, 102)
ARNILA	oncogenic	Enhances SOX4, induce EMT	Migration, metastasis	(103)
SNHG12	oncogenic	Regulation of MMP13 expression	Correlated with lymph node involvement, metastasis	(104)
H19	oncogenic	Inhibits BIK and NOXA (Bcl2 family) and reduces apoptosis	poor prognosis, chemo-resistance	(105)
POU3F3	Tumor suppressor	Regulates Caspase-9	promotes proliferation and inhibits apoptosis	(106)
RMST	Tumor suppressor	regulation of mRNA/proteins	prevent migration and invasion	(107)
NEF	Tumor suppressor	downregulates miR-155	increased migration and invasion	(108)
Airn	Tumor suppressor	suppresses the Wnt/β-catenin/mTOR/PI3K pathway	Increase migration and invasion	(109)

PRC2, Polycomb Repressive Complex 2; MMP, matrix metalloproteinase.

HOTAIR induces migration and invasion of TNBC cell lines and was the first lncRNA to act as a marker of metastasis (83, 84). There are numerous reports of a strong association between lymph node metastasis in TNBC and HOTAIR expression in tissues (84–87) and circulation (88). Moreover, patients with upregulated HOTAIR tend to have poor survival rates. As lncRNAs are highly stable in biological fluids (89–93), detection of HOTAIR in blood samples from patients with TNBC can provide prognostic information and facilitate monitoring of treatment response. LncRNA ARNILA has a key role in TNBC locoregional invasion and metastasis. It can promote EMT by competitively binding to miR-204, which normally inhibits SOX4 expression. Upregulated ARNILA functions as an oncogenic lncRNA because, by sequestering miR-204, it frees SOX4 to induce EMT and metastatic spread. Moreover, silencing of ARNILA effectively inhibits cancer invasion, migration, and metastasis (103). From tumor supressor lncRNAs were described RMST, NEF and Airn. LncRNA RMST was identified as an lncRNA with very low expression levels in TNBC cells compared with healthy tissues. RMST localizes to the cytoplasm and is believed to exert its functions via regulation of mRNA or proteins. When RMST is over-expressed, the number of TNBC cells in the G1/G0 phase increases and the number of migrating cells reduces, indicating that RMST can prevent TNBC cell migration and invasion. In conclusion, RMST warrants further investigation as a potential biomarker and therapeutic tool in patients with TNBC (107).

## Interactions Among lncRNA, miRNA, and mRNA in TNBC

Fully mature miRNAs interact with complementary regions in the 3’-UTRs of target mRNA transcripts, referred to as miRNA recognition elements (MREs). The consequence is the destruction of targets that are a perfect match or inhibition of translation when the target is a partial match (110).

Every miRNA can target hundreds of mRNAs and contribute to various functions and numerous networks. Individual miRNA mutations alone do not frequently lead to abnormal phenotypes but are strongly related to several network parameters, including cellular milieu, network topology, genomic position, and evolutionary age (111). There is extensive cooperation among miRNAs expressed in various functional contexts. The targeting of a single mRNA by multiple miRNAs leads to selective and specific regulation of its functions. Two co-operating miRNAs can bind to mRNA and form a thermodynamically stable triplex with synergistically increased effectiveness (112, 113). A single miRNA can simultaneously influence several mRNA targets participating in the same process, coordinated with other miRNAs, upstream transcription factors, and downstream mRNA targets. These elements are interconnected and form functional network modules with the general form “transcription factor/miRNA/target gene” (114, 115).

Some examples of lnRNAs-miRNA interactions in TNBC can involve lnRNAs as MALAT1, AWPPH and NEF. MALAT1 maps to chromosome 11q13 and is upregulated in invasive non-small cell lung carcinoma (94). It exhibits an intranuclear location and induces the PI3k/AKT/mTOR and Wnt/β-catenin pathways in multiple neoplasms, including TNBC (95). Zuo et al. suggests that silencing MALAT1 could decrease cell proliferation, migration, and invasion, inducing cell cycle arrest in the G0/G1 phase. Moreover, their study proves that, in TNBC, MALAT1 acts as a sponge for miR-129-5p, thereby promoting disease progression (98). Also, upregulated AWPPH expression is correlated with TNBC (116). A study discovered an association between AWPPH and miR-21, which together promote cell proliferation in TNBC and contribute to the development of chemoresistance. As miR-21 and lncRNA AWPPH are upregulated in TNBC and interact to function in cell proliferation, regulation, and chemoresistance, they have potential for use as blood biomarkers for early diagnosis (102). LncRNA NEF is an lncRNA that has been studied in TNBC cells and patients, demonstrating that NEF downregulates miR-155 and that altered NEF expression leads to high miR-155 levels in plasma. Overexpression of miR-155 combined with low lncRNA NEF levels is a profile that can differentiate patients with TNBC from controls. Downregulated NEF, alongside high levels of circulating miR-155, is correlated with increased migration and invasion of malignant cells. NEF over-expression also downregulates miR-155 expression, and miR-155 over-expression has no effect on lncRNA NEF, suggesting that NEF acts to directly downregulate miR-155 (108).

The arrangement of miRNAs in networks and the number of their interconnections with other molecules has a major influence on their functionality. The connection points of networks comprise miRNAs or mRNAs; those with high numbers of “synapses” are referred to as “hubs” and have major roles in regulating the whole network. mRNAs are situated in hub positions with higher densities of target sites and are evolutionarily selected as major points of direct miRNA-mediated suppression (117).

Several miRNAs associate in cancer networks with p73, a p53 family member. These interactions include inhibition of p73 by miRNAs, downregulation of miRNAs by p73, or modulation of the proteins that stabilize p73 by miRNAs. On the other hand, p73 regulates transcription of crucial miRNA genes having several attractive features in the context of cancer therapy including TNBC: 1) p73, unlike p53, is less often mutated in neoplastic disease; 2) its isoform, Tap73, inhibits all cancer features, developing responsiveness to standard radio- and chemotherapies; and 3) p73 replaces p53 functions, inducing the same axes and regulating stress-response pathways and retaining their function when p53 is dysfunctional (69, 118).

## ceRNA

Approximatively 20,000 protein-coding genes have been identified in the human genome, a large number of which are coated with MREs (119). Pseudogenes are genomic loci that are similar to known genes but generally do not encode functional proteins and are defined as “nonfunctional,” “junk,” or “evolutionary relics” (120, 121). Due to their strong resemblance to miRNA sequences, pseudogenes can act as competitors for the same pool of miRNAs through sets of conserved MREs (122).

Salmena et al. were the first to propose the concept of competitive endogenous RNA (ceRNA), which encompasses all types of transcript: mRNA, tRNA, rRNA, lncRNA, pseudogene RNA, and circular RNA. In the ceRNA hypothesis, the traditionally envisaged process is reversed: That is, mRNAs are considered to influence miRNAs, suggesting that mRNAs sharing multiple MREs cross-talk and compete for miRNA binding. The result is reduced miRNA recognition and consequent diminished miRNA activity (102, 123). MREs are “artificial transcripts” comprising repeats of miRNA responsive elements, also termed “miRNA-sponges,” and are considered “the letters” of a “hidden RNA language” represented by ceRNAs. This language represents a complex system of interactions between different RNA species that manages gene expression regulation (124).

Another hypothesis states that ceRNA cross-talk cells center on a simple set of interactions between one miRNA and two target mRNAs (ceRNAs). The most important factors in this model are 1) the rate of transcription and degradation of miRNA and ceRNAs and 2) the rates of association, dissociation, and degradation of the complex. The ideal balance of ceRNA-mediated cross-regulation is obtained when all components of the system are equally represented. Secondary communication in ceRNETs may cause overexpression of the effect of a perturbation at any level of the network. As ceRNAs are key players in gene regulation, they are considered to represent an evolutionary mechanism (125, 126). It is generally accepted that ceRNA networks could be the next candidates to serve as prognostic biomarkers and possibly targets for new therapies in TNBC.

Based on recent topics of research focus, which involve genomic sponges and interactions among them, databases (ceRDB, starBAse, lnCeDB, LncACTdb, HumanViCe) have been created to archive all available information. Relationships are predicted virtually and validated experimentally (125).

## The Interactive Axis of Tumorigenesis, Proliferation, and Progression in TNBC

Many lncRNAs have been proposed as ceRNAs for miRNAs. In TNBC, ARF6 overexpression causes loss of MiR-145, resulting in promotion of cell invasion. LincRNA-RoR, a regulator of reprogramming, is a ceRNA for miR-145, leading to competitive inhibition with ARF6, and loss of mature miR-145 expression (126–130).

Another important discovery is circular RNAs (circRNAs), which are ceRNAs created by the direct ligation of the 5′ and 3′ ends of linear RNA. Circular forms are transitional in the course of RNA splicing, exhibit superior stability relative to linear RNAs, and could consequently serve as effective miRNA sponges (131).

Direct regulatory relationships in these networks manifest as correlations among genes regulated by the same transcription factors, transcription factors with their individual correlated targets, or incidental correlations between gene expression levels. It has become clear that co-expression networks created in this way do not accurately represent the underlying regulatory processes and do not retain many of the properties associated with biological networks (132).

Recently, weighted gene co-expression network analysis (WGCNA) has been developed as a data mining method for studying ceRNA networks in TNBC. WGCNA was performed using mRNAs and lncRNAs extracted from mammary tissue of patients with triple-negative disease and the information subsequently correlated with Ki-67 status, using TCGA database. The *RAD51AP1* and *TYMS* mRNAs were discovered to play a major role in overall survival of patients with TNBC based on analysis of this ceRNA network. The results of that study demonstrate that specific mRNAs and lncRNAs are part of a ceRNA network in TNBC, which could serve as target for treatment of patients with TNBC in the future (133).

A study of 116 TNBC and 11 normal tissues from TCGA analyzed integrated expression profiles, including data on miRNAs, lncRNAs, and mRNAs in a WGCNA to identify the features of dysregulated genes. Seven key molecules (*AKAP12*, *FOS*, *EMX2OS*, *MYCNOS*, *RP11-542B15.1*, hsa-miR-9, and hsa-miR-183) were overexpressed and correlated with poor patient outcomes; however, overexpression of hsa-miR-145, LINC00461, RP11-576D8.4, and RP11-496D24.2 was correlated with better OS (134).

An lncRNA-miRNA-mRNA regulatory axis was also hypothesized as positively correlated with TNBC in a ceRNA, in which five molecules (*TERT*, *TRIML2*, *PHBP4*, miR-1-3p, and miR-133a-3p) were significantly associated with prognosis in patients with TNBC (135).

Study of lncRNA-mRNA-miRNA axis interactions in a cohort of 1441 patients with TNBC from TGCA database showed that nine lncRNAs, including HOTTIP, ST7-AS2, LINC00491, ST7-OT4, NLGN1-AS1, ELFN2, C1orf143, C10orf126, and C8orf31, were associated with TNBC TNM stage, suggesting that they have oncogenic roles in TNBC. Further, differentially expressed lncRNAs (DElncRNAs) were found to be involved in progression of TNBC with four lncRNAs, RBMS3-AS3, C8orf31, LINC00452, and LINC00200, showing great potential as prognostic predictors in TNBC (136).

In a study of 155 DElncRNAs, DEmRNAs, and DEmiRNAs, OSTN-AS1 was identified as related to immunologic function and immune-related marker co-expression and proposed as a novel, possibly immune-related, prognostic marker (137).

## Biomarkers

A biomarker is a molecule that, ideally, is easy to detect and offers credible information on diagnosis, prognosis, or other disease parameters (138). MiRNAs are considered possible strong biomarkers in TNBC and can be evaluated in tissue specimens and as circulating miRNAs, where the latter present new opportunities for identification of powerful biomarkers in TNBC (139, 140).

### Tissue miRNAs as Biomarkers in TNBC

MiRNAs useful as biomarkers can be identified singly or as part of a group of miRNAs all implicated in TNBC, referred to as miR-signatures. Examples of miRNAs useful as independent markers and also included in various miR-signatures include miR-10b, miR-155, and miR-21. Further, these molecules are dysregulated in numerous neoplasms in addition to TNBC (71, 141).

An miR-signature comprising 11 miRNAs (miR-21, miR-125b, miR-155, miR-181a, miR-181b, miR-10b, miR-195, miR-31, miR-183, miR-130a-3p, miR-451a) can distinguish between TNBC tumor and normal breast tissue (139). Further, in a cohort of 11 patients with TNBC, tissue biopsies were obtained prior to systemic therapy. A signature comprising three miRs (miR-200b-3p, miR-190a, and miR-512-5p) was demonstrated as being associated with complete pathological response to different treatment protocols (140). Another study in a cohort of 173 TNBC cases (up to 50 years old) discovered an additional miR-signature (miR-16, miR-655, miR-421, miR-374a, miR-374b, miR-497, miR-125b, miR-374a, miR-155, miR-16, miR-125b) that could function as a predictive biomarker for OS and disease-free survival (116). Further, miR-148a and miR-629-3p are associated with lung metastases, and miR-141 was found to promote brain metastasis (67, 142, 143). The miR-10 family is also implicated in TNBC progression and metastasis (144).

High expression of miR-95-3p is significantly correlated with decreased OS and relapse-free survival in patients treated with anthracycline-based chemotherapy, and another five-miRNA signature (including let-7d-3p, miR-30a-3p, miR-30c-5p, miR-128-3p, and miR-95-3p) was validated as a novel prognostic and predictive biomarker in TNBC, predicting patient response to anthracycline-based chemotherapy (145). Further, two miR-signatures were detected in a study of 173 TNBC cases. The first four-miR signature (miR-155, miR-16, miR-374a, and miR-125b) is associated with poor OS rate. The second also comprises four miRs (miR-155, miR-27a, miR-30e, and miR-493) and is linked to BC classification based on ER/PR/HER2/basal cytokeratin/EGFR status as well as separation of cases into low- and high-risk categories (50) with the ability to predict patient response to therapy with the two most common systemic protocols used to treat TNBC (anthracycline or anthracycline plus taxanes) (146).

### Circulating miRNAs as Biomarkers in TNBC

**Figure 1:**
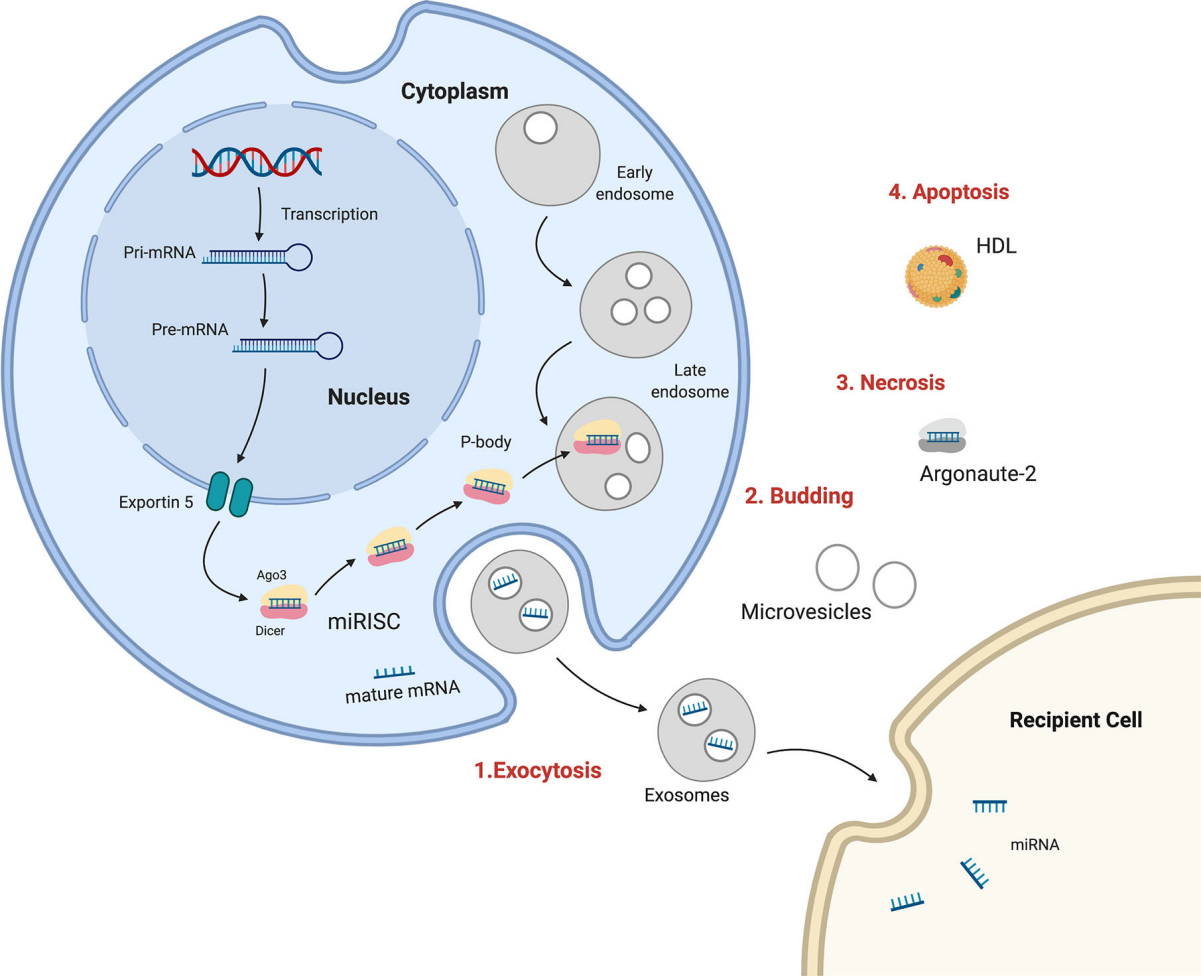
Sources and routes of circulating mRNAs. 1. Exosomal secretion- Pri-miRNA is transcribed , processed by Drosha and transported in cytoplasm by Exportin5, where is integrated in RISC complex and target mRNA in exosomes which are released in human fluids. 2. Budding from plasma membrane and forming microvesicles. 3. Necrosis, with the releasing of AGO-miRNA complexes. 4. Apoptosis, bounding high-density lipoproteins (HDL).

Many recent studies have described circulating miRNAs as promising diagnostic, prognostic, or predictive biomarkers for BC. The processes of miRNA biogenesis and maturation occur in the cell nucleus and cytoplasm, and miRNAs can be discharged out of cells from the cytoplasm to become extracellular circulating miRNAs. There are multiple mechanisms for miR transportation within organisms, including 1) wrapped in large apoptotic bodies, 2) in HDL or LDL lipoprotein complexes, 3) in microvesicles or smaller exosomes, and 4) in an AGO protein complex (147). Exosomes and lipid vesicles have critical roles in cell–cell communication. Cell-free circulating miRNAs can be detected and isolated from the microenvironment as well as from different body fluids, such as plasma, serum, saliva, urine, seminal fluid, breast milk, and cerebrospinal fluid (148) (**Figure 1**).

The processes used to validate miRNAs as biomarkers in TNBC have strengths and weaknesses. The great advantage of miRNAs in the pre-analytical phase is that they can be detected in biological fluids, require a minimally invasive procedure for collection, and are stable under various conditions (repetitive freeze-thaw, extreme pH values, up to 24 h at room temperature, or for long periods at 70°C) (149). Problems with validation of miRNA biomarkers include several biases related to the daily habits of patients (physical activity, smoking, diet, kidney pathology, and circadian rhythm), sample collection, and handling (149–153). Real-time quantitative polymerase chain reaction (RT-qPCR) is the standard analytical technique used to assess circulating miRNAs; however, some alternative platforms, including other PCR-based assays, microarray, and next-generation sequencing (NGS) can also be used. Validation can be affected in the analytical phase by noncirculating miRNA contamination (blood cells, skin, and activated platelets) and by hemolysis (154–157). Finally, issues of concern in the postanalytical phase include a lack of a standardized protocols and procedures (158).

### New Trends Relating to Circulating miRNAs as Potential Diagnostic Biomarkers for Early Detection, Prediction, and Prognosis of TNBC

IVarious studies have confirmed the importance of analysis of miRNAs as a noninvasive approach for screening and management of BC molecular subtypes (**Table 3**). Downregulation of miR-195-5p and miR-495 has value as a prospective circulating surrogate molecular marker for early detection of luminal or TNBC (171). Another seven-serum miRNA panel (miR-489-3p + miR-199a-3p + miR-195-5p + miR-15a-5p + miR-7-5p + let-7c-5p + let-7i-5p) can serve as a diagnostic marker for patients with TNBC (172). Further, a panel of nine miRNAs (miR-15a, miR-18a, miR-107, miR-133a, miR-139-5p, miR-143, miR-145, miR-365, and miR-425) is a potential blood-based multimarker test for BC detection (173). Moreover, another five circulating miRNA combination (miR-1246, miR-1307-3p, miR-4634, miR-6861-5p, and miR-6875-5p) can detect TNBC with a sensitivity of 97.3%, specificity of 82.9%, and accuracy of 89.7% for BC in the test cohort. Furthermore, this combination can detect early stage BC (sensitivity 98.0% for stage 0) (174).

**Table 3:** Circulating miRNA as diagnostic, predictive, or prognostic biomarkers.

Type of miR	Expression	Biological fluid	TNBC stage	Relevance	Special features	References
miR-10b	Upregulated	Plasma/ serum	Early	Lymph node status	Identification of bone metastasis	(159–161)
miR-199a-5p	Downregulated	Plasma	T0–T1	Tumor growth	Level inversely proportional to disease stage	(161, 162)
miR-105	Upregulated	Plasma	Early	Predicts survival and choice of treatment, promotes metastasis	Promotes cisplatin resistance	(163)
miR-16	Downregulated	Plasma	Advanced	Marker for overall survival		(161, 162)
miR-489	Upregulated	Plasma				(164)
miR-93-3p	Upregulated	Plasma	Early	Predicts survival, informs choice of treatment	Promotes cisplatin resistance	(161, 163)
miR-195-5p	Upregulated	Plasma	Early	Early detection		(161)
miR-495	Downregulated	Plasma	Early	Early detection		(165)
miR-18a	Upregulated	Serum		Choice of treatment	Involved in paclitaxel resistance	(166)
miR-18b	Upregulated	Serum	Early	Diagnosis, prognosis		(161, 166)
miR-143	Downregulated	Serum	Early	Diagnosis		(166)
miR-153	Upregulated	Serum				(166)
miR-155	Upregulated	Serum	Early	Diagnosis, lymph node status, response to therapy	Possibly contributes to chemoresistance	(166, 167)
miR-373	Upregulated	Plasma	Early	Diagnosis, lymph node status	Correlated with CD44 expression	(159, 167)
miR-17	Downregulated	Serum	Advanced	Predicts recurrence, informs choice of treatment		(167, 168)
miR-19b	Upregulated	Serum				(168)
miR-200c	Downregulated	Plasma	Metastatic	Lymph node status, distant metastasis	Decreased levels of miR-200c were only observed in metastatic tumors	(160, 169)
miR-34a/c	Downregulated	Plasma	All stages	Predictive for OS	miR-34c is an independent factor predicting worse prognosis	(167, 170)

In stage II and III TNBC patients, the overexpression of many circulating miRNAs (hsa-miRNA-188-5p, hsa-miRNA-1202, hsa-miRNA-4281, hsa-miRNA-1207-5p, hsa-miRNA-4270, hsa-miRNA-1225-5p, hsa-miRNA-642b-3p, hsa-miRNA-1290, hsa-miRNA-3141, miRNA-127-3p, miRNA-148b, miRNA-409-3p, miRNA-652, and miRNA-801) has been identified and validated (175). Serum miR-21 expression level is associated with lymph node metastasis and high Ki-67 expression in TNBC (*p* < 0.01) (176).

Some circulating plasma miRNAs can accurately discriminate TNBC from non-TNBC. In TNBC, miR-199a-5p, miR-21, and miR-16 are downregulated (AUC: 0.88, 0.80, and 0.87, respectively), and their levels are restored to normal after surgery. MiR-489, miR-200b, miR-193b, miR-125b, miR-105, miR-93 3p are upregulated in TNBC (AUC: 0.99, 0.88, 0.91, 0.97, 0.93, and 0.66, respectively), which has been validated in diagnostic settings. In addition, circulating miR-34a is downregulated in patients with both TNBC and HR^+^/HER2^-^ BC tumors unresponsive to neoadjuvant chemotherapy compared to those with responsive tumors (AUC = 0.59) (177).

In a meta-analysis of 21 relevant studies (2510 patients) exploring the prognostic value of miRNAs in TNBC by measuring miR expression levels in tumor or blood samples, six miRNAs were assessed: miR-155, miR-21, miR-27a/b, miR-374a/b, miR-210, and miR-454. The results indicated that decreased expression of miR-155 is associated with reduced OS (adjusted hazard ratio (HR) = 0.58, 95% confidence interval (CI): 0.34–0.99; crude HR = 0.67, 95% CI: 0.58–0.79). High miR21 expression was also predictive of reduced OS (crude HR = 2.50, 95% CI: 1.56–4.01), and elevated levels of miR-27a/b, miR-210, and miR-454 expression were associated with shorter OS, and levels of miR-454 and miR-374a/b expression were associated with DFS (178).

### LncRNAs as Potential Biomarkers in TNBC

In a prospective observational study using frozen tissue sections, 165 TNBC samples and 33 paired normal breast tissues were analyzed using transcriptome microarrays. An integrated mRNA-lncRNA signature, based on eight mRNAs and two lncRNAs (HIST2H2BC and SNRPEP4), was evaluated. This signature is more feasible for predicting 2-year recurrence-free survival than classic prognostic factors (AUC 0.714) and can accurately predict clinical outcomes and benefit from of taxane chemotherapy in patients with TNBC (179). Expression levels of Linc00339 in several BC cell lines were compared with those in a normal mammary gland epithelial cell line, and higher expression of miR-377-3p indicates longer OS in patients with TNBC. Hyperexpression of miR-377-3p produces a delay in TNBC cell growth by regulating cell cycle distribution and apoptosis and miR-377-3p regulates HOXC6 expression, influencing Linc00339-mediated TNBC proliferation. Hence, a Linc00339/miR-377-3p/HOXC6 axis influences TNBC progression and may be a promising therapeutic target for TNBC treatment (180).

Measurement of HIF1A-AS2 expression in 86 TNBC specimens, 30 non-TNBC specimens, and 30 adjacent mammary specimens demonstrated that it is upregulated in TNBC tissues compared with non-TNBC tissues, leading to the conclusion that HIF1A-AS2 expression is associated with OS in patients with TNBC (181).

Another study analyzed the expression of the lncRNA, *HOTAIR*, in 163 cases of TNBC and found that its expression in tumor tissues is strongly correlated with lymph node metastasis and has a direct strong association with androgen receptor (*AR*) expression. These data suggest the involvement of HOTAIR in regulation of AR-mediated pathways, leading to its proposal as a prognostic marker correlated with new therapeutic strategies for patients with the LAR type of TNBC (93). Furthermore, plasma urothelial carcinoma associated 1 (UCA1) levels are significantly elevated in patients with TNBC, indicating that this molecule may act as a specific biomarker for TNBC diagnosis (182).

A recently discovered lncRNA, hepatocellular carcinoma upregulated EZH2-associated lncRNA (HEIH), is overexpressed in TNBC tissues and cell lines compared with adjacent normal mammary tissues and a normal mammary epithelial cell line. Downregulation of HEIH inhibits TNBC cell proliferation and promotes apoptosis by regulating the miR-4458/SOCS1 axis. HEIH is also involved in clinical progression (183).

Comparison between the genome-wide methylation profiles in peripheral blood DNA from 233 patients with TNBC and 231 controls led to the identification and validation of increased methylation at cg06588802 in the long intergenic noncoding RNA, LINC00299 in patients with TNBC compared with controls, suggesting that hypermethylation of *LINC00299* in peripheral blood may constitute a useful circulating biomarker for TNBC (184).

## Discussion

The paradigm proposed for the origin of cancer related to its hallmarks involves the highly dynamic accumulation of mutations and chromosomal genetic changes occurring at different times and influenced by epigenetic mechanisms.

Translating current knowledge of TNBC into oncological practice involves identification of new biomarkers/molecules/assays, which reveal new pathways or novel ways to understand these diseases. EMT is a current research focus with the metastatic process involving multiple genes/pathways: for example, ETS, RAS, integrins, WNT/beta-catenin, SRC, and Notch (185–191).

Cancer stem cells express phenotypic plasticity, which is a major factor in tumor malignancy, and miRNAs are involved in the maintenance of stemness in TNBC (192–196). Specifically, miRNA-31 promotes mammary stem cell expansion and tumorigenesis by suppressing WNT signaling antagonists (197).

LncRNAs are also central players in the battle against malignant diseases. LncRNAs contribute to regulatory networks by various mechanisms. Nuclear lncRNAs may be considered to have epigenetic functions of regulation or to act as guides by recruiting chromatin modification factors to cytogenetic loci.

Studies of miRNAs and lncRNAs can also improve information regarding the molecular classification of TNBC. Recently a novel TNBC classification system, the FUSCC classification, was established by integrating the expression profiles of mRNAs and lncRNAs (198). Therefore, it is essential to explore the mechanisms and interactions involved in the regulatory networks, including lncRNA, miRNA, mRNA, genetic mutations, and epigenetic changes in TNBC. A new cross-talk pattern, represented by the ceRNA hypothesis, in which lncRNAs can serve as a ceRNAs involved in regulation of mRNA expression and also including miRNAs, represents a new perspective regarding ncRNA interactions.

Possible tissue biomarkers have been identified in numerous studies, particularly miRNAs, singly or associated in specific signatures, relevant for TNBC subtypes, prognosis, and prediction of treatment efficacy.

The importance of circulating lncRNAs and miRNAs as robust noninvasive biomarkers, particularly for the diagnosis of TNBC, is demonstrated by emerging evidence. A major issue for biomarkers can arise if circulating RNA profiles do not resemble miRNAs within the tumor. An analysis of 210 plasma miRNAs suggested that they may match those in tumors although other studies report differences in expression between serum and tumor miRNAs, attributable to only a small proportion of miRNAs being released from the tumor into the circulation. Differentially expressed miRNA and target genes are suggested to form complex interaction networks, affecting many biological processes (199).

## Conclusions and Future Perspectives

LncRNA, miRNA, and mRNA interactions in cancer and the molecular mechanisms involved in these interactions correlated with tumorigenesis and cancer progression remain undefined. Future biomarkers in TNBC may be represented by genomic signatures developed into clinically accepted tests for the early detection and clinical management of patients with TNBC.

Circulating miRNAs and lncRNAs are also promising tests for early detection, prognosis, and monitoring treatment effects. LncRNA-miRNA-mRNA interaction networks provide opportunities for further experimental studies and improvement of biomarker predictions for developing novel therapeutic approaches in TNBC.

## Author Contributions

All authors listed have made a substantial, direct, and intellectual contribution to the work and approved it for publication.

## Conflict of Interest

The authors declare that the research was conducted in the absence of any commercial or financial relationships that could be construed as a potential conflict of interest.
